# Social vulnerabilities in head-neck melanoma care: A retrospective cohort study in the United States

**DOI:** 10.1016/j.jdin.2024.05.011

**Published:** 2024-07-30

**Authors:** Lillian McCampbell, David Jun Fei-Zhang, Daniel Chelius, Ling-Lun Bob Hsia, Robert Dellavalle, Jill D’Souza, David Bentrem, Jeffrey Wayne, Jeffrey Rastatter, Anthony Sheyn

**Affiliations:** a Department of Otolaryngology-Head and Neck Surgery, University of Tennessee Health Science Center, Memphis, Tennessee; b Department of Otolaryngology, Northwestern University Feinberg School of Medicine, Chicago, Illinois; c Department of Otolaryngology-Head and Neck Surgery, Pediatric Thyroid Tumor Program and Pediatric Head and Neck Tumor Program, Baylor College of Medicine, Texas Children’s Hospital, Houston, Texas; d Department of Dermatology, Medical University of South Carolina, Charleston, South Carolina; e Department of Dermatology, University of Colorado Anschutz Medical Campus, Aurora, Colorado; f Division of Pediatric Otolaryngology, Children’s Hospital of New Orleans and Louisiana State University, New Orleans, Los Angeles; g Department of Surgery, Northwestern University Feinberg School of Medicine, Chicago, Illinois; h Division of Pediatric Otolaryngology, Ann & Robert H. Lurie Children’s Hospital of Chicago, Chicago, Illinois; i Department of Pediatric Otolaryngology, Le Bonheur Children’s Hospital, Memphis, Tennessee; j Department of Pediatric Otolaryngology, St. Jude Children’s Research Hospital, Memphis, Tennessee

**Keywords:** head and neck, melanoma, melanoma treatment, social determinants of health

## Abstract

**Background:**

Studies addressing social determinants of health (SDH) in head-neck melanomas (HNM) have only assessed socioeconomic factor impact but not a wider scope of SDH.

**Objective:**

Utilizing the Social Vulnerability Index (SVI), to assess the influence of specific SDH and their quantifiable associations with HNM management disparities across the varied community contexts in the United States.

**Methods:**

This retrospective cohort study analyzed adults diagnosed with HNM from 1975 to 2017 from the Surveillance, Epidemiology, and End Results Program database.

**Results:**

A total of 374,138 HNM in adults from 1975 to 2017 were assessed for disparities affiliated with increasing overall vulnerability/SVI scores and SDH themes. For several melanoma subtypes, higher social vulnerability significantly decreased odds (lowest for amelanotic, odds ratio 0.74; 95% confidence interval, 0.63-0.86) for indicated surgery, increased odds of indicated radiation (highest for epithelioid cell, 1.44; 1.08-1.96), and advanced staging on first presentation (highest for acral lentiginous, 1.13; 1.01-1.27). Household composition, followed by socioeconomic status and minority-language status contributed significantly to the overall trend.

**Limitations:**

Limitations include unknown cause of death and SVI score calculation based on county of residency.

**Conclusions:**

This investigation highlights significant detrimental trends in HNM management with overall social vulnerability while showcasing the quantifiable associations of specific SDH themes on HNM-disparities.

**Capsule Summary**

• Head-neck melanoma patients observed significantly decreased odds of receiving surgery and increased odds of radiation, alongside having advanced staging on preliminary presentation as social vulnerability increased.
• These observations provide delineations about which social determinants associate with disparities in management, setting the foundations for targeted public health policy and prospective implementations.

## Introduction

As melanoma continues to increase in incidence by 5% annually,^1^ several efforts have been made to understand factors influencing this rise. Namely, investigations encompassing UV exposure, awareness of the disease, genetics, and skin color have all been empirically focused for their contributions to higher frequencies of melanoma diagnosis.^2^^,^^3^

Among these drivers, social determinants of health (SDH) have often been investigated in this dynamic of melanoma management. In particular, patients with higher socioeconomic status (SES) have been observed to have higher incidence of melanoma, whereas lower SES patients have been observed to have worse outcomes of melanoma.^4^, ^5^, ^6^, ^7^ In addition, race-ethnicity have shown different prevalence rates among melanoma subtypes of superficial spreading, which predominantly affect Caucasian patients, and acral lentiginous, which showed significantly higher prevalence rates in patients with skin of color compared to patients who were non-Hispanic White.^8^, ^9^, ^10^, ^11^, ^12^ However, sparse literature exists about varied SDH beyond the effects of SES and racial identity, especially among melanomas diagnosed in the head and neck, which make up 25% of melanoma diagnoses yearly. Furthermore, the interactions of how such SDH play out in generalizable, sociodemographic contexts have been seldom investigated.

**Fig 1:**
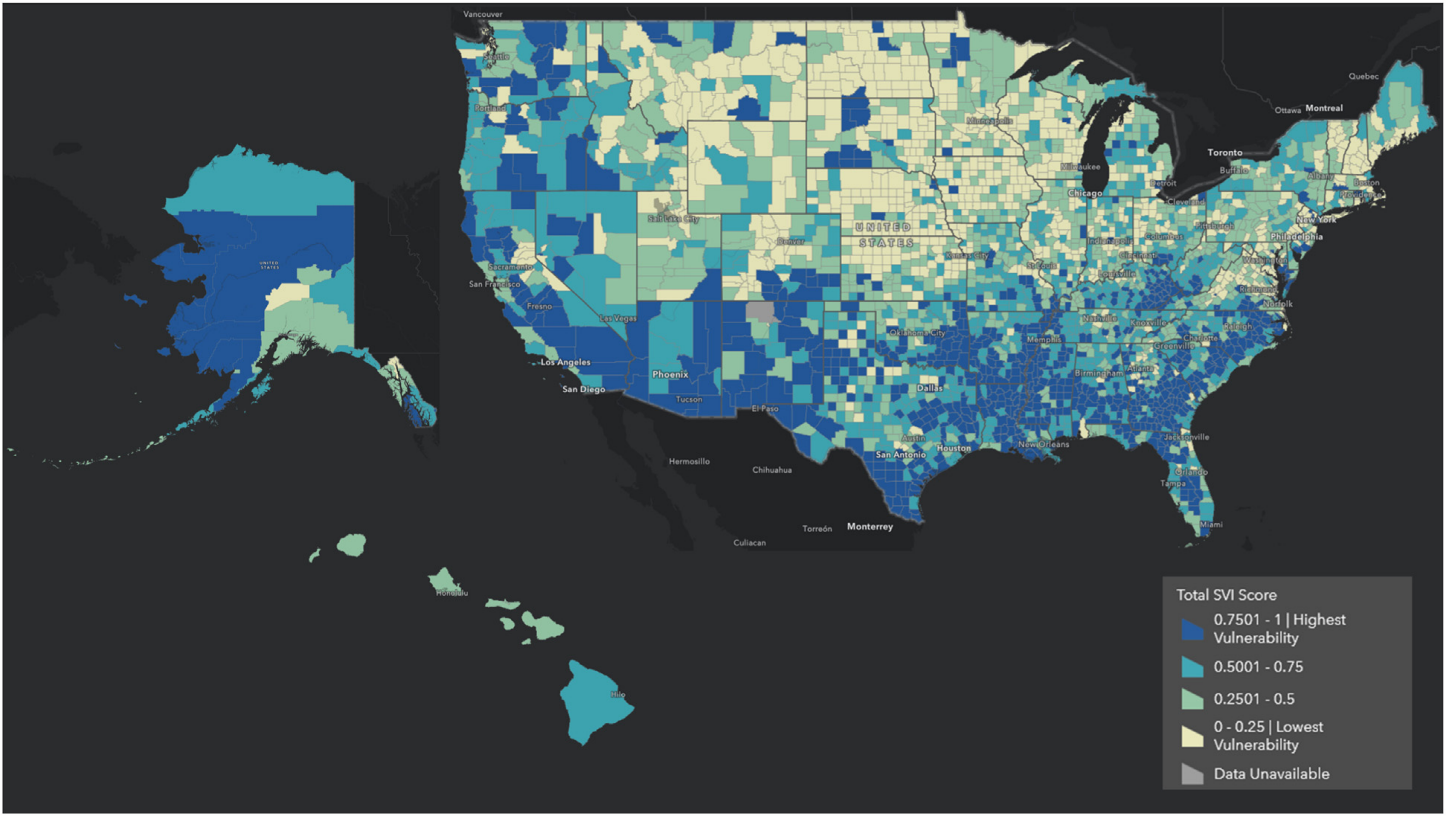
Total social vulnerability by social vulnerability index (SVI) scores across the United States.

The social vulnerability index (SVI) is a US Census-based tool created by the Centers for Disease Control and Prevention, featuring 15 social factors measured across the nation. These factors are grouped into categories of SES, minority & language status (ML), household composition (HH), and housing & transportation (HT). (Fig 1) SVI has seen rising utility in wider health care aspects of understanding cancer disparities. These include prior associations with gastrointestinal and lung cancer outcomes, as well as our prior work on pediatric head-neck cancers.^13^, ^14^, ^15^, ^16^ However, its utility has yet to be fully explored regarding the diagnosis and treatment of HNM. Thus, our study aims to apply the SVI and its various SDH themes to assess the interactional relationship between increasing SDH-vulnerability and prognostic and management disparities of HNM to elucidate their real-world impact in the United States.

## Materials and methods

This retrospective cohort study follows the strengthening the reporting of observational studies in epidemiology (STROBE) reporting guidelines. No previous institutional review board or ethics committee approval was needed due to the databases queried consisting of publicly available, deidentified data.

### Databases and population definitions

The SVI was queried for ranked scores among 15 social factors within 4 SDH themes of SES (poverty, unemployment, income level, and high school diploma status), ML (minoritized racial and ethnic group [American Indian and Alaska Native, Asian, Black or African American, American Indian and Alaska Native, Asian, Native Hawaiian or other Pacific Islander, other, and multiracial] (per the US Census) and proficiency with English), HH (household members aged ≥65 and ≤17 years, disability status, single-parent status), and HT (multiunit structure, mobile homes, crowding, no vehicle, group quarters) as well as total composite scores. These SDH themes are differentially weighed to form the total composite score and are assigned different weights based on the designated area’s sociodemographic and census data. These differential weights are not made publicly available due to restrictive access to identifier information. Total and SDH-theme scores are based on relative social vulnerabilities of a particular census tract among all 72,158 US Census tracts. These scores range from 0 to 1, with 0 representing the lowest social vulnerability and 1 representing the highest.

The National Cancer Institute Surveillance, Epidemiology, and End Results Program (SEER) database comprises nationwide data sets with various patient variables, including staging and treatment receipt characteristics. The advanced staging was characterized by SEER-designated variables labeled as, stage IV, distant (expansion) (compared with in-situ, localized, or regional or stage I-III), or distal (expansion) (compared with local, regional [expansion]) and recoded under American Joint Committee on Cancer, 6th edition (AJCC-6) classifications. Primary surgery occurrence indicates whether patients received surgery for their primary malignant neoplasm. Primary radiation occurrence indicates whether patients received radiation therapy (external beam) for their primary malignant neoplasm.

#### Population definitions

SEER was queried for adult patients (20+ years) diagnosed with head and neck melanomas from 1975 to 2017. Melanoma diagnoses derived from primary sites of the head and neck were extracted using the International Classification of diseases for Oncology, 3rd edition (ICD-O-3) topographic codes: C44.0-44.9. Melanoma subtypes were extracted using histology ICD-O-3 codes: 8720-8790. These classification schemes were validated by the SEER data administrators for this specific data set utilized.

**Fig 2:**
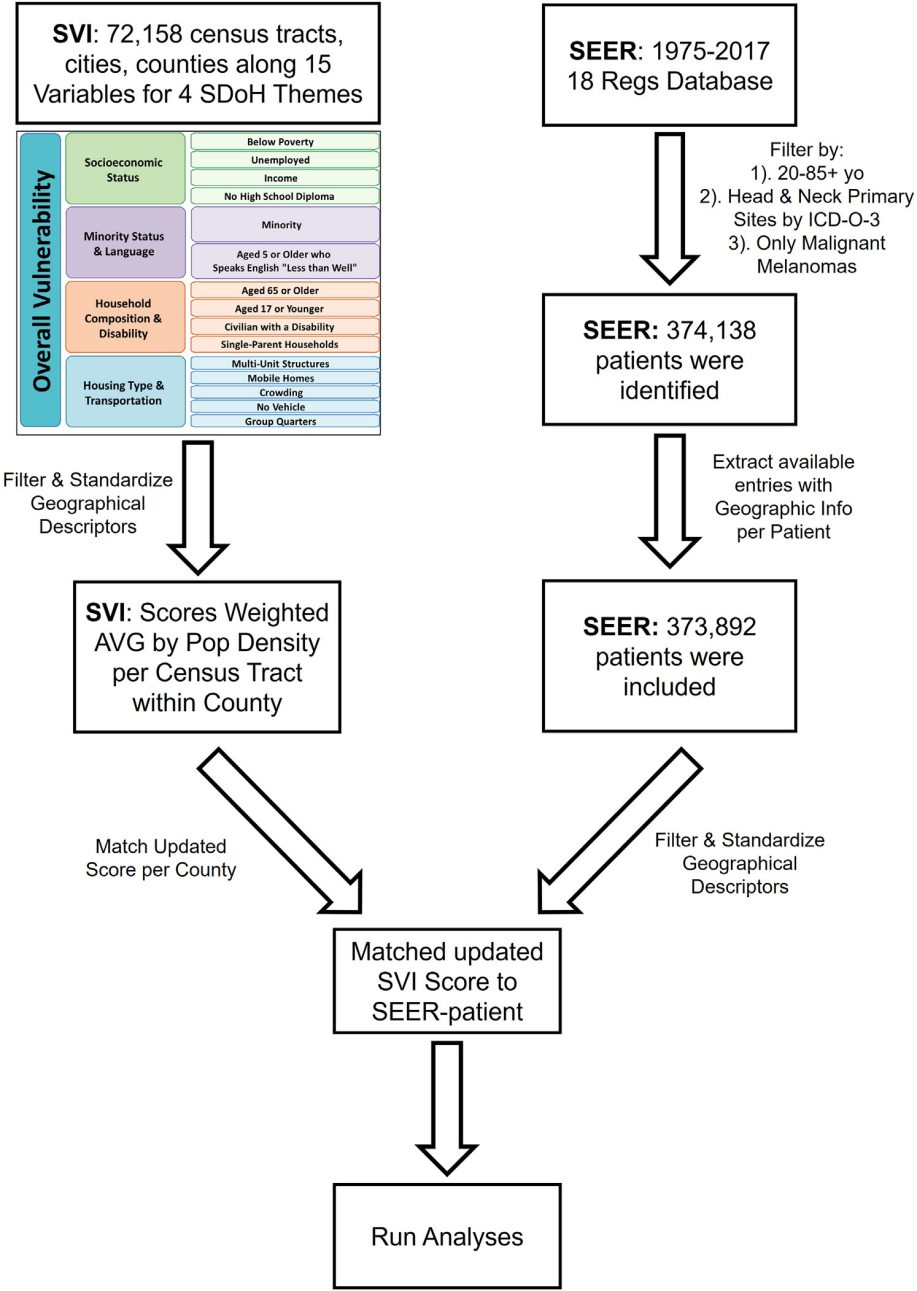
Schematic workflow of data processing.

The SVI scores were abstracted and matched to patients in the SEER database on county of residence at the time of diagnosis. County-assigned scores were generated by weighted score means per population density of each census tract within the county. A workflow diagram of this database merging process can be found in Fig 2.

### Statistical analysis

Total and subtheme SVI scores were split into relative equivalently sampled quintiles based on actual scores within each type of melanoma. These quintiles were then used as ranked, discrete variate levels (with reference level set to the lowest vulnerability quintile) within univariate logistic regression models assessing associated occurrences of whether patients received primary surgical treatment, primary radiation treatment, or possessed advanced staging on preliminary presentation. Univariate logistic regressions were utilized to preserve the differential relationship between SDH themes and total SVI scores, as internal, dynamically-weighted means are utilized to generate total SVI scores. Statistical significance was set to *P* < .05. Two-sided *P* values were reported for analyses. Analyses were conducted in R version 4.2.3 (R Project for statistical computing).

## Results

Among 374,138 head-neck melanoma patients identified, 373,892 were included in the study based on the presence of necessary analytic variables. The most represented histology subtypes of HNM were not otherwise specified (n = 174,447, 46.7%), superficial spreading (n = 126,012, 33.7%), and lentigo maligna (n = 26,678, 7.1%). Total CDC-SVI scores ranged from 0.00 to 0.95; SES sub-scores ranged from 0.00 to 0.98; ML sub-scores ranged from 0.00 to 0.95; HH sub-scores ranged from 0.09 to 0.97; HT sub-scores ranged from 0.05 to 0.94. Additional cohort characteristics are represented in Table I.

**Table I:** Head-neck melanoma patient characteristics by increasing social vulnerability

Characteristic	N	Total SVI score				
		0.000-0.199, n = 4120 (1.1%)	0.200-0.399, n = 96,041 (26%)	0.400-0.599, n = 18,9824 (51%)	0.600-0.799, n = 79,567 (21%)	0.800-0.999, n = 4340 (1.2%)
Age, y	373,892					
20-44		811 (20%)	20,811 (22%)	38,091 (20%)	13,476 (17%)	652 (15%)
45-64		1,758 (43%)	36,889 (38%)	72,525 (38%)	29,803 (37%)	1573 (36%)
65-84		1384 (34%)	32,828 (34%)	67,225 (35%)	30,645 (39%)	1796 (41%)
85+		167 (4.1%)	5513 (5.7%)	11,983 (6.3%)	5643 (7.1%)	319 (7.4%)
Sex	373,892					
Male		2354 (57%)	53,652 (56%)	108,202 (57%)	46,815 (59%)	2540 (59%)
Female		1766 (43%)	42,389 (44%)	81,622 (43%)	32,752 (41%)	1800 (41%)
Race	373,892					
White		3952 (96%)	92,180 (96%)	174,972 (92%)	71,041 (89%)	3732 (86%)
Unknown		82 (2.0%)	2243 (2.3%)	7486 (3.9%)	3212 (4.0%)	235 (5.4%)
Hispanic		72 (1.7%)	1128 (1.2%)	4597 (2.4%)	4177 (5.2%)	285 (6.6%)
Asian or Pacific Islander		6 (0.1%)	203 (0.2%)	1477 (0.8%)	465 (0.6%)	11 (0.3%)
Black		2 (<0.1%)	199 (0.2%)	964 (0.5%)	500 (0.6%)	33 (0.8%)
Native American		6 (0.1%)	88 (<0.1%)	328 (0.2%)	172 (0.2%)	44 (1.0%)
Region	373,892					
Midwest		323 (7.8%)	17,708 (18%)	20,690 (11%)	887 (1.1%)	0 (0%)
Northeast		1520 (37%)	32,106 (33%)	22,444 (12%)	2816 (3.5%)	0 (0%)
South		1540 (37%)	12,144 (13%)	36,396 (19%)	16,536 (21%)	1461 (34%)
West		737 (18%)	34,083 (35%)	110,294 (58%)	59,328 (75%)	2879 (66%)
Histological type	373,892					
Acral lentiginous melanoma, malignant		12 (0.3%)	732 (0.8%)	1,824 (1.0%)	962 (1.2%)	56 (1.3%)
Amelanotic melanoma		14 (0.3%)	369 (0.4%)	807 (0.4%)	391 (0.5%)	18 (0.4%)
Desmoplastic melanoma, malignant		23 (0.6%)	903 (0.9%)	2201 (1.2%)	943 (1.2%)	60 (1.4%)
Epithelioid cell melanoma		11 (0.3%)	285 (0.3%)	401 (0.2%)	195 (0.2%)	6 (0.1%)
Lentigo maligna melanoma		451 (11%)	7677 (8.0%)	13,838 (7.3%)	4488 (5.6%)	224 (5.2%)
Malignant melanoma in giant pigmented nevus		8 (0.2%)	204 (0.2%)	595 (0.3%)	258 (0.3%)	13 (0.3%)
Malignant melanoma in junctional nevus		2 (<0.1%)	254 (0.3%)	544 (0.3%)	361 (0.5%)	9 (0.2%)
Malignant melanoma, not otherwise specified		1818 (44%)	44,990 (47%)	86,019 (45%)	39,254 (49%)	2366 (55%)
Malignant melanoma, regressing		14 (0.3%)	407 (0.4%)	884 (0.5%)	356 (0.4%)	21 (0.5%)
Mixed epithelioid and spindle cell melanoma		10 (0.2%)	147 (0.2%)	298 (0.2%)	220 (0.3%)	4 (<0.1%)
Nodular melanoma		224 (5.4%)	6506 (6.8%)	13,756 (7.2%)	6964 (8.8%)	357 (8.2%)
Spindle cell melanoma, not otherwise specified		27 (0.7%)	858 (0.9%)	2051 (1.1%)	1124 (1.4%)	66 (1.5%)
Superficial spreading melanoma		1506 (37%)	32,709 (34%)	66,606 (35%)	24,051 (30%)	1140 (26%)
Primary surgery performed	371,548					
No surgery		154 (3.8%)	3017 (3.2%)	7301 (3.9%)	4592 (5.8%)	290 (6.7%)
Surgery		3926 (96%)	92,393 (97%)	181,324 (96%)	74,539 (94%)	4012 (93%)
Radiation therapy performed	373,892					
No therapy		4079 (99%)	94,926 (99%)	187,353 (99%)	78,323 (98%)	4234 (98%)
Therapy		41 (1.0%)	1115 (1.2%)	2471 (1.3%)	1244 (1.6%)	106 (2.4%)
Vital status on last follow-up	373,892					
Alive		3338 (81%)	68,030 (71%)	132,648 (70%)	55,009 (69%)	2788 (64%)
Dead		782 (19%)	28,011 (29%)	57,176 (30%)	24,558 (31%)	1552 (36%)

### Treatment receipt with social vulnerability

Across ICD-O-3 disease classes, increasing total CDC-SVI vulnerability was associated with decreased odds of receiving surgical intervention for those with amelanotic melanoma (odds ratio [OR], 0.74, 95% confidence interval [CI], 0.63-0.86; *P* < .001), lentigo maligna melanoma (OR, 0.84, 95% CI, 0.80-0.87; *P* < .001), malignant melanoma in junctional nevus (OR, 0.77, 95% CI, 0.60-0.97; *P* = .026), malignant melanoma not otherwise specified (NOS) (OR, 0.82, 95% CI, 0.81-0.84; *P* < .001), nodular melanoma (OR, 0.91, 95% CI, 0.86-0.96; *P* = .001), spindle cell melanoma NOS (OR, 0.89, 95% CI, 0.80-1.00; *P* = .044), and superficial spreading melanoma (OR, 0.89, 95% CI, 0.87-0.91; *P* < .001) **(**Table II).

**Table II:** Treatment receipt with increasing social vulnerability

Histology Type∗	Characteristic	Surgery			Radiation therapy		
		OR	95% CI	*P*	OR	95% CI	*P*
Acral lentiginous melanoma, malignant	Total	0.88	0.76-1.02	.089	1.08	0.90-1.30	.405
Socioeconomic status		0.88	0.76-1.02	.090	0.99	0.83-1.19	.929
Minority-language status		0.86	0.74-0.99	.045	1.15	0.96-1.38	.139
Household composition		0.97	0.84-1.12	.709	0.99	0.83-1.19	.929
Housing-transportation		0.96	0.83-1.11	.606	1.11	0.92-1.33	.267
Amelanotic melanoma	Total	0.74	0.63-0.86	< .001	1.14	0.96-1.37	.143
Socioeconomic status		0.78	0.67-0.91	.001	1.18	0.99-1.42	.067
Minority-language status		0.84	0.73-0.97	.020	0.89	0.74-1.07	.205
Household composition		0.82	0.71-0.95	.008	1.27	1.06-1.53	.010
Housing-transportation		0.79	0.68-0.91	.001	1.03	0.87-1.24	.711
Desmoplastic melanoma, malignant	Total	0.88	0.78-1.00	.051	1.04	0.97-1.11	.313
Socioeconomic status		0.87	0.77-0.98	.027	1.07	1.00-1.15	.056
Minority-language status		0.91	0.80-1.03	.119	0.96	0.89-1.03	.264
Household composition		0.95	0.84-1.08	.446	1.05	0.98-1.13	.139
Housing-transportation		0.92	0.82-1.05	.209	1.02	0.95-1.09	.614
Epithelioid cell melanoma	Total	0.82	0.61-1.08	.154	1.44	1.08-1.96	.011
Socioeconomic status		0.89	0.67-1.17	.392	1.17	0.89-1.55	.262
Minority-language status		0.68	0.50-0.91	.009	1.47	1.11-2.01	.007
Household composition		1.22	0.93-1.64	.158	0.91	0.69-1.19	.497
Housing-transportation		0.89	0.67-1.17	.396	1.27	0.96-1.69	.093
Lentigo malignant melanoma	Total	0.84	0.80-0.87	< .001	1.03	0.92-1.16	.594
Socioeconomic status		0.83	0.80-0.87	< .001	1.01	0.90-1.13	.859
Minority-language status		0.84	0.80-0.87	< .001	0.95	0.85-1.07	.409
Household composition		0.97	0.93-1.01	.165	0.98	0.87-1.10	.724
Housing-transportation		0.96	0.92-1.00	.037	1.09	0.97-1.23	.139
Malignant melanoma in giant pigmented nevus	Total	1.06	0.81-1.39	.692	1.00	0.64-1.57	> .999
Socioeconomic status		0.93	0.70-1.21	.579	1.17	0.75-1.87	.495
Minority-language status		1.06	0.81-1.39	.687	1.00	0.64-1.57	> .999
Household composition		0.92	0.70-1.21	.569	1.23	0.79-1.99	.363
Housing-transportation		1.40	1.06-1.89	.019	0.77	0.47-1.21	.261
Malignant melanoma in junctional nevus	Total	0.77	0.60-0.97	.026	1.13	0.56-2.44	.723
Socioeconomic status		0.60	0.45-0.77	< .001	1.29	0.64-2.94	.476
Minority-language status		0.73	0.57-0.93	.010	1.29	0.64-2.94	.476
Household composition		0.95	0.75-1.19	.638	1.49	0.73-3.69	.280
Housing-transportation		1.21	0.96-1.54	.101	0.88	0.41-1.79	.723
Malignant melanoma, not otherwise specified	Total	0.82	0.81-0.84	< .001	1.06	1.03-1.09	< .001
Socioeconomic status		0.80	0.78-0.81	< .001	1.10	1.07-1.13	< .001
Minority-language status		0.86	0.85-0.87	< .001	0.96	0.94-0.99	.009
Household composition		0.91	0.90-0.93	< .001	1.13	1.10-1.16	< .001
Housing-transportation		0.89	0.87-0.90	< .001	0.99	0.97-1.02	.640
Malignant melanoma, regressing	Total	0.87	0.70-1.07	.196	0.98	0.74-1.30	.892
Socioeconomic status		0.77	0.62-0.96	.018	0.96	0.72-1.27	.781
Minority-language status		0.98	0.79-1.20	.820	1.00	0.76-1.33	.99
Household composition		0.85	0.68-1.05	.132	1.23	0.93-1.66	.151
Housing-transportation		0.99	0.80-1.22	.908	0.96	0.72-1.27	.781
Mixed epithelioid and spindle cell melanoma	Total	0.95	0.70-1.29	.751	1.07	0.80-1.43	.651
Socioeconomic status		0.91	0.67-1.23	.533	1.07	0.80-1.43	.651
Minority-language status		1.05	0.77-1.42	.767	0.96	0.72-1.28	.777
Household composition		0.95	0.70-1.29	.751	1.02	0.77-1.37	.875
Housing-transportation		0.91	0.67-1.23	.533	1.22	0.91-1.66	.180
Nodular melanoma	Total	0.91	0.86-0.96	.001	1.09	1.04-1.14	< .001
Socioeconomic status		0.90	0.85-0.95	< .001	1.16	1.11-1.22	< .001
Minority-language status		0.87	0.82-0.92	< .001	0.95	0.91-1.00	.030
Household composition		1.03	0.98-1.09	.271	1.15	1.10-1.21	< .001
Housing-transportation		0.92	0.87-0.98	.006	1.01	0.96-1.05	.777
Spindle cell melanoma, not otherwise specified	Total	0.89	0.80-1.00	.044	1.02	0.93-1.11	.740
Socioeconomic status		0.91	0.82-1.02	.118	1.00	0.91-1.10	.960
Minority-language status		0.86	0.77-0.97	.010	0.90	0.82-0.98	.020
Household composition		1.06	0.95-1.19	.289	1.08	0.99-1.19	.092
Housing-transportation		0.89	0.79-0.99	.039	0.98	0.89-1.07	.645
Superficial spreading melanoma	Total	0.89	0.87-0.91	< .001	1.06	0.99-1.12	.079
Socioeconomic status		0.90	0.88-0.92	< .001	1.10	1.03-1.17	.003
Minority-language status		0.86	0.84-0.88	< .001	0.92	0.86-0.98	.006
Household composition		1.05	1.02-1.07	< .001	1.15	1.08-1.22	< .001
Housing-transportation		0.96	0.94-0.98	.001	1.01	0.95-1.08	.684

Univariate logistic regressions across increasing levels of total and SDH-theme SVIs were performed to assess dichotomic outcomes of primary surgery or radiation therapy receipt of a patient being treated for head-neck melanoma.

∗ By American Joint Committee on Cancer, 6th edition (AJCC-6).

The SVI-themes of SES, ML, HT, and HH vulnerability contributed to these overall social vulnerability trends by logistic regression analyses. For amelanotic melanoma, malignant melanoma NOS, and superficial spreading melanoma, increasing SES, ML, HT, and HH vulnerability were associated with decreased odds of receiving surgery. For lentigo maligna melanoma and nodular melanoma, increasing SES, ML, and HT vulnerability, but not HH vulnerability, were associated with decreased odds of receiving surgery. For malignant melanoma in junctional nevus increasing SES, and ML vulnerability, but not HH and HT vulnerability, were associated with decreased odds of receiving surgery. For spindle cell melanoma NOS increasing ML and HT vulnerability, but not SES and HH vulnerability, were associated with decreased odds of receiving surgery (Table II).

Across ICCC disease classes, increasing total CDC-SVI vulnerability was associated with increased odds of receiving radiation for those with epithelioid cell melanoma (OR, 1.44, 95% CI, 1.08-1.96; *P* = .011), malignant melanoma NOS (OR, 1.06, 95% CI, 1.03-1.09; *P* < .001), and nodular melanoma (OR, 1.09, 95% CI, 1.04-1.14; *P* < .001) (Table II).

For malignant melanoma NOS and nodular melanoma, increasing SES, ML, and HH vulnerability, but not HT vulnerability, were associated with increased odds of receiving radiation. For epithelioid cell melanoma, increasing ML vulnerability, but not SES, HH, and HT vulnerability, were associated with increased odds of receiving radiation (Table II).

### Advanced staging on presentation with social vulnerability

Across ICCC disease classes, increasing total CDC-SVI vulnerability was associated with increased odds of having advanced stage at first presentation for those with acral lentiginous melanoma malignant (OR, 1.13, 95% CI, 1.00-1.27; *P* = .050), malignant melanoma NOS (OR, 1.06, 95% CI, 1.04-1.08; *P* < .001), and nodular melanoma (OR, 1.06, 95% CI, 1.02-1.10; *P* = .006) (Table III).

**Table III:** Late staging on first presentation with increasing social vulnerability

Histology type∗	Characteristic	OR	95% CI	*P*
Acral lentiginous melanoma, malignant	Total	1.13	1.01-1.27	.049
Socioeconomic status		1.10	0.97-1.24	.127
Minority-language status		1.13	1.00-1.27	.055
Household composition		1.08	0.96-1.22	.198
Housing-transportation		1.08	0.96-1.22	.192
Amelanotic melanoma	Total	1.05	0.91-1.20	.527
Socioeconomic status		1.00	0.87-1.15	.948
Minority-language status		0.93	0.81-1.07	.299
Household composition		1.11	0.97-1.28	.138
Housing-transportation		1.10	0.96-1.26	.187
Desmoplastic melanoma, malignant	Total	1.08	0.96-1.20	.186
Socioeconomic status		1.06	0.95-1.19	.270
Minority-language status		1.01	0.90-1.13	.849
Household composition		1.05	0.94-1.18	.351
Housing-transportation		0.97	0.87-1.09	.645
Epithelioid cell melanoma	Total	1.17	0.92-1.51	.201
Socioeconomic status		1.08	0.85-1.39	.521
Minority-language status		1.14	0.89-1.46	.292
Household composition		0.98	0.77-1.26	.898
Housing-transportation		0.99	0.77-1.26	.917
Lentigo maligna melanoma	Total	1.05	0.91-1.20	.511
Socioeconomic status		1.09	0.95-1.25	.218
Minority-language status		0.96	0.83-1.10	.533
Household composition		1.17	1.02-1.35	.023
Housing-transportation		0.95	0.83-1.09	.444
Malignant melanoma in giant pigmented nevus	Total	0.93	0.64-1.36	.722
Socioeconomic status		1.26	0.86-1.88	.237
Minority-language status		0.74	0.49-1.09	.130
Household composition		1.25	0.86-1.87	.249
Housing-transportation		0.60	0.37-0.90	.012
Malignant melanoma in junctional nevus	Total	0.93	0.54-1.58	.783
Socioeconomic status		1.07	0.63-1.86	.788
Minority-language status		1.00	0.58-1.71	> .999
Household composition		1.16	0.68-2.04	.589
Housing-transportation		0.92	0.53-1.58	.768
Malignant melanoma not otherwise specified	Total	1.06	1.04-1.08	< .001
Socioeconomic status		1.11	1.09-1.14	< .001
Minority-language status		0.97	0.94-0.99	.002
Household composition		1.13	1.11-1.16	< .001
Housing-transportation		0.98	0.96-1.00	.099
Malignant melanoma-regressing	Total	1.10	0.89-1.36	.384
Socioeconomic status		1.04	0.84-1.29	.715
Minority-language status		1.15	0.93-1.43	.193
Household composition		1.19	0.96-1.49	.104
Housing-transportation		1.01	0.81-1.25	.937
Mixed epithelioid and spindle cell melanoma	Total	0.99	0.78-1.26	.941
Socioeconomic status		1.02	0.81-1.30	.861
Minority-language status		0.87	0.68-1.10	.254
Household composition		1.17	0.92-1.50	.191
Housing-transportation		0.92	0.72-1.17	.483
Nodular melanoma	Total	1.06	1.02-1.10	.006
Socioeconomic status		1.09	1.05-1.14	< .001
Minority-language status		1.03	0.99-1.07	.214
Household composition		1.07	1.03-1.11	.001
Housing-transportation		0.99	0.95-1.03	.543
Spindle cell melanoma not otherwise specified	Total	1.02	0.92-1.13	.698
Socioeconomic status		1.03	0.93-1.14	.554
Minority-language status		1.03	0.94-1.14	.494
Household composition		1.04	0.94-1.15	.453
Housing-transportation		0.98	0.89-1.08	.652
Superficial spreading melanoma	Total	1.05	0.99-1.11	.104
Socioeconomic status		1.10	1.04-1.16	.001
Minority-language status		0.91	0.86-0.96	.001
Household composition		1.13	1.07-1.19	.000
Housing-transportation		1.03	0.97-1.09	.316

Univariate logistic regressions across increasing levels of total and SDH-theme SVIs were performed to assess binary outcomes of having stage IV or distal expression in comparison to stage I-III or non-distal expression of head-neck melanoma disease.

∗ By American Joint Committee on Cancer, 6th edition (AJCC-6).

For malignant melanoma NOS increasing SES, ML, and HH vulnerability, but not HT vulnerability, were associated with increased odds of having advanced stage at first presentation. For nodular melanoma increasing SES and HH vulnerability, but not ML and HT vulnerability, were associated with increased odds of having advanced stage at first presentation (Table III).

## Discussion

To our knowledge, there is sparse literature utilizing SVI as a measure of SDH of head and neck melanoma on a community level for specifically analyzing treatment delivery and staging. Increasing overall social vulnerability, measured by SVI, showed decreased odds for indicated surgery, increased odds for indicated radiation, and increased odds for advanced stage at first presentation. The value in this study is the ability to look at SVI and melanoma subtypes with a granular view and identify areas of head and neck melanoma disparities.

It is known that higher SES correlates with higher number of melanoma while lower SES correlates with later stage presentation melanoma.^4^^,^^5^^,^^17^, ^18^, ^19^ Jiang et al^20^ conducted a systemic review investigating the relationship between SES and incidence and mortality of melanoma. They discussed SES in terms of both income and lifestyle factors. Lifestyle factors were occupation, environmental exposure, UV light exposure, marital status, travel practices, hobbies, smoking habits, and activity. They found that married marital status led to earlier presentation of melanoma while widowed individuals were more likely to present with late-stage disease and had an increased risk of death from melanoma. In addition, they found that those living in deprived areas, smokers, body mass index greater than 30, and low educational attainment were more likely to present with later stage melanoma. These findings reinforce the discovery that higher SVI correlates to advanced staging on first presentation. In a study by Yan et al,^12^ acral lentiginous melanoma was specifically studied. Using SEER data, they found that patients in the lowest quintile of SES were 3 times more likely to have stage IV disease, and stage I was more common within the higher SES quintiles. Our data show that acral lentiginous melanoma had the highest risk of presenting with advanced stage, with an increased odds ratio of 1.13.

Treatment of melanoma can vary based on location, tumor diameter, and thickness. Our study showed that patients with higher SVI had a decreased chance to be treated with surgery. Zell et al^21^ corroborated these claims by finding that higher SES patients were more likely to receive surgery, and that surgery had decreased risk of death. Melanoma cases in African Americans were adjusted for age, sex, histologic subtype, stage, anatomic site, treatment with surgery, radiation therapy, chemotherapy and SES and still had worse survival rates than non-Hispanic White patients. Al-Qurayshi et al^22^ further discovered that those with low income that did receive surgery as treatment, were more likely to be treated by low-volume surgeons in high-risk community or rural hospitals. In addition, they found that Blacks and Hispanics had longer hospital stays than their White melanoma counterparts and had increased cost of health care for treatment. These studies that investigate outcomes of surgical excision of melanoma indicate that even though patients with higher SVI are less likely to receive surgery as treatment, when they are treated with surgery, they still have less favorable outcomes than patients with lower SVI.

Surgery is the standard of care first-line treatment for local melanoma. If the disease progresses to metastatic melanoma, radiation can be used alone or as an adjuvant therapy to surgery. Our studies showed that higher SVI groups were more likely to receive indicated radiation treatment. This is likely because of the increased chance for these patients to present with advanced disease, thus needing radiation therapy or adjuvant radiation along with surgical excision. Berk et al^23^ comments on the use of radiation for local melanoma when surgery is not a viable option, or unavailable. They specifically discuss head and neck mucosal melanomas, and the primary control of melanomas with radiation, although local melanomas tended to have the best success with surgery and adjuvant radiation. Phillips et al^24^ commented on the impact of COVID-19 on late-stage presentation of melanomas. With halting of in-person appointments, more patients were presenting with more advanced disease of all types. Phillips et al^24^ detailed that improving prevention and early detection are the largest contributors to improving outcomes of melanoma. Health literacy is the largest modifiable factor pertaining to early detection. Unfortunately, those with limited health literacy are more likely to present with late-stage disease and thus require radiation as adjunct treatment to surgical resection.

Our data found that increasing social vulnerability showed increased usage of indicated radiation, inferring associations of social vulnerability and poorer prognosis. To our knowledge, the literature on the effect of social disparities on outcomes of melanoma treated with radiation, is limited aside from studies of treatment of melanoma metastasis or as adjuvant therapy.^25^^,^^26^ It is important to conduct further studies to determine whether melanoma treated with radiation outcomes are differentiated among those of different social vulnerability statuses.

### Strengths and limitations

This study utilizes the largest sample size of head and neck melanoma patients. In addition, granular data analysis was made possible by subdividing melanoma and SVI into sub-grouped analysis. Unfortunately, this study was limited by the variables provided by the SEER database. Cause of death of patients was unknown. In addition, SVI subcategories do not measure all SDH, or all factors that increase patients’ social vulnerability. In addition, SVI scores were calculated based on county of residence, not individual patient information. Use of regional SVI as a proxy for personal social vulnerability may lead to over or under representation in certain cases. In addition, given the scope of US Census data utilized by the SVI mainly encompassed demographic trends from 2000 to 2018 based on its usage of 5-year summarized information spanning this time, its applicability to melanoma patients diagnosed before 2000 may be limited.

## Conclusion

In this study, we showed that with increasing social vulnerability, head and neck melanoma patients were less likely to receive indicated surgery, more likely to receive indicated radiation, and more likely to present with advanced disease. These findings are consistent with the literature published and additionally offer a granular view of what SDH play larger roles in specific melanoma subtypes. These data provide a rare opportunity for providers to identify how and exactly where social vulnerabilities affect patients. Awareness of this information is the first step toward creating change and providing opportunities to utilize knowledge of patient SVI to bridge the disparity gap across sociodemographic groups receiving different melanoma management.

## Conflict of interest

Dr Chelius reported receiving a coordinator stipend from the American Academy of Otolaryngology outside the submitted work. No other disclosures are reported. Authors McCampbell and Fei-Zhang and Drs Hsia, Dellavalle, D’Souza, Bentrem, Wayne, Rastatter, and Sheyn have no conflicts of interest to declare.
